# Pure proton therapy for skull base chordomas and chondrosarcomas: A systematic review of clinical experience

**DOI:** 10.3389/fonc.2022.1016857

**Published:** 2022-11-25

**Authors:** Menglin Nie, Liying Chen, Jing Zhang, Xiaoguang Qiu

**Affiliations:** ^1^ Department of Radiation Oncology, Beijing Tiantan Hospital, Capital Medical University, Beijing, China; ^2^ Laboratory of Pathology, Hebei Cancer Institute, The Fourth Hospital of Hebei Medical University, Shijiazhuang, Hebei, China

**Keywords:** proton therapy, chordoma, chondrosarcoma, skull base, efficacy, toxicity

## Abstract

**Background:**

Skull base chordoma and chondrosarcoma are exceptionally rare bone tumors with high propensity for local recurrence. Different postoperative radiation modalities are often used to improve the clinical efficacy. Proton therapy (PT) might be among the most promising ones because of the unique ballistic characteristics of high-energy particles. However, previous meta-analysis often included studies with combined radiation techniques. No systematic review to date has directly analyzed the survival and toxicity of pure PT for these two types of malignancies.

**Methods:**

By following the PRISMA guidelines, a systematic search of three databases was conducted. Articles were screened and data were extracted according to a prespecified scheme. R 4.2.0 software was used to conduct the meta-analysis. Normal distribution test was used for the incidence rate of each subgroup.

**Results:**

A total of seven studies involving 478 patients were included in this analysis. The quality of included articles ranged from moderate to high quality. All patients were histopathologically diagnosed with chordoma or chondrosarcoma, and the follow-up time of the cohort ranged from 21 to 61.7 months. For PT planning, the median target volume ranged from 15 cc to 40 cc, and the administered median dose varied from 63 to 78.4 Gy_RBE_ at 1.8–2.0 Gy_RBE_ per fraction. The 1-, 2-, 3-, 5-, and 7-year local control and overall survival rates were 100%, 93%, 87%, 78%, and 68%, and 100%, 99%, 89%, 85%, and 68%, respectively. The late grade 3 or higher toxicities were reported in only two involved articles.

**Conclusions:**

Until now, medical centers worldwide have exerted PT to improve outcomes of skull base chordomas and chondrosarcomas. PT not combined with other radiation modalities showed favorable local control and survival with a low incidence of severe radiation-induced toxicities, which manifests promising clinical benefits. However, high-quality evidence is still limited, requiring future clinical trials and prospective studies in selected patients.

## Introduction

Chordoma and chondrosarcoma are slowly progressive, locally aggressive bone tumors with extremely low incidence (1). The common locations often include the skull base, as well as the extracranial skeleton, mostly in the sacrococcygeal area and spine. Skull base chordomas mainly arise from remnants of the notochord in the clivus of sphenoid bone, usually presenting with typical symptoms, such as headache and diplopia (2). Chordoma is mainly divided into three common categories, namely, conventional, chondroid, and dedifferentiated histopathologic subtypes (3). Chondrosarcomas are rare malignant cartilaginous neoplasms comprising 6% of all skull base primary malignant bone tumors (4, 5). Both of these rare pathological tumors require maximal debulking resection so as to achieve optimal clinical outcomes, whereas a bloc clean surgical resection is difficult to achieve because of the complex anatomy of the skull base. In addition, due to the intrinsic aggressive pathological features, tumors tend to relapse and progress frequently even after achieving optimal surgical resection (1). As a result, postoperative adjuvant therapies are crucial to achieve favorable local control and long-term survival of these patients (6, 7).

To date, radiotherapies with different modalities and radioactive particles have been developed. Radiotherapy used curatively or postoperatively seems to be an effective measure to precisely eliminate the residual or subclinical lesions, reduce local recurrence, and improve prognosis (8–10).

Conventional photon radiotherapy (CRT) has been employed since the last century and is the most accessible radiation therapy modality. Despite the fact that CRT can deliver nearly 60–70 Gy to a highly conformed tumor target, dose escalation to greater than 70 Gy might be required for chordomas and chondrosarcomas to achieve better tumor control (11, 12). However, tumors located in the skull base represent a challenge for CRT because adjacent critical neurologic structures, such as optic pathways, brainstem, and cervical spinal cord, limit the prescription dose. Charged particles such as protons were then explored to improve the biological effect of radiotherapy, as well as to protect organs at risk and minimize both early and late radiation-related toxicities (13, 14). Compared to photon therapy, the ballistic characteristics of proton beams loaded with sharp kinetic energy allow more dose escalation to the precise target while limiting the exposure dose of adjacent critical structures (15, 16).

To date, medical centers worldwide have gathered more information about the effects of PT used as either radical or adjuvant therapy in skull base chordomas and chondrosarcomas (17–19). However, access to PT is still limited and much more costly compared with conventional photon therapy; thus, the included patients in some existing systematic reviews were not purely receiving PT. In this systematic review, we analyzed the clinical outcomes and potential toxicities of skull base chordomas and chondrosarcomas after PT-only plannings without combination of any other radiation modalities. A better insight into delivering proton beams alone in skull base chordomas and chondrosarcomas should be achieved, and the accuracy together with reliability of published research should be clarified.

## Methods

### Search strategy

The systematic search was conducted on three electronic literature databases (1997–present), namely, PubMed, the Cochrane Library, and MEDLINE, to identify articles appropriate for this study. The Medical Subject Headings (MeSH) terms and keywords used for searching were as follows: (“chordoma” [MeSH Terms] OR “chordomas” [All Fields]) AND (“skull base neoplasms” [MeSH Terms] OR “skull base” [All Fields] OR “clivus” [All Fields] OR “cranial” [All Fields] OR “intracranial” [All Fields] OR “atlas” [All Fields]) AND (“proton therapy” [MeSH Terms] OR “proton therapies” [All Fields] OR “proton beam therapy” [All Fields] OR “proton beam radiation therapy” [All Fields]) AND (“radiotherapy” [MeSH Terms] OR “radiotherapies” [All Fields] OR “radiation therapy” [All Fields] OR “radiation therapies” [All Fields]) AND (“survival” [All Fields] OR “mortality” [All Fields] OR “prognosis” [All Fields] OR “prognostic factor” [All Fields] OR “recurrence” [All Fields]).

### Selection criteria

The review was conducted in compliance with the Preferred Reporting Items for Systematic reviews and Meta-Analysis (PRISMA) guidelines and recommendations [1]. Only English-language articles were included. All retrieved articles were screened by two reviewers (MN and LC). The inclusion criteria were as follows (1): primary or recurrent chordoma or chondrosarcoma located in the skull base location, (2) managed by proton-only radiotherapy after surgical resection or biopsy, and (3) the observation indicator included either survival outcomes and/or toxicity incidence. Either local control (LC) or overall survival (OS) could be considered as survival outcomes. The exclusion criteria were the combined photon–proton radiotherapy and median follow-up of less than 1 year. Rare presentations or metastases due to clival chordoma should also be excluded.

### Metadata extraction

The full text of all eligible articles should be available and then a data extraction form was made. All the observation indicators, including authors and year of study, study period, location of the study conducted, trial design, cohort size, median age and follow-up, pathological diagnosis, surgical resection, median target volume, proton radiotherapy regimen, survival outcomes, and toxicity, were extracted from each included article.

### Statistical techniques

R 4.2.0 software (R-4.2.0, 64 bit, The Cochrane Collaboration, Oxford, UK) was used to make a single-arm meta-analysis. Normal distribution test was performed before further analysis. All the *W*-values were close to 1 and *p*-value > 0.05. The Cochran *Q* test and *I²* statistics were used to assess the heterogeneity. The forest plots were used to show the results of a single study and summary analysis. The funnel plots and Egger’s linear regression test were conducted to analyze the bias assessment of all studies. Sensitivity analysis was also used to analyze the publication bias.

### Certainty, quality, and bias assessments

To assess the quality of included articles, the Newcastle–Ottawa Scale (NOS) (20) was used. Items for assessment included representativeness of the exposed cohort, whether it was histopathologically confirmed, whether the follow-up was long enough for outcomes to occur, whether all important data were cited in the report, and whether the outcome was correctly ascertained. The Grading of Recommendations, Assessment, Development and Evaluations (GRADE) (21) criteria were used to assess the certainty of the results based on the information of the included studies. Publication bias was assessed using funnel plots and Egger’s regression. Asymmetry in plots indicated publication bias (22), and Egger’s regression test provided a statistical verification of funnel plot asymmetry.

## Results

### Search strategy

**Figure 1:**
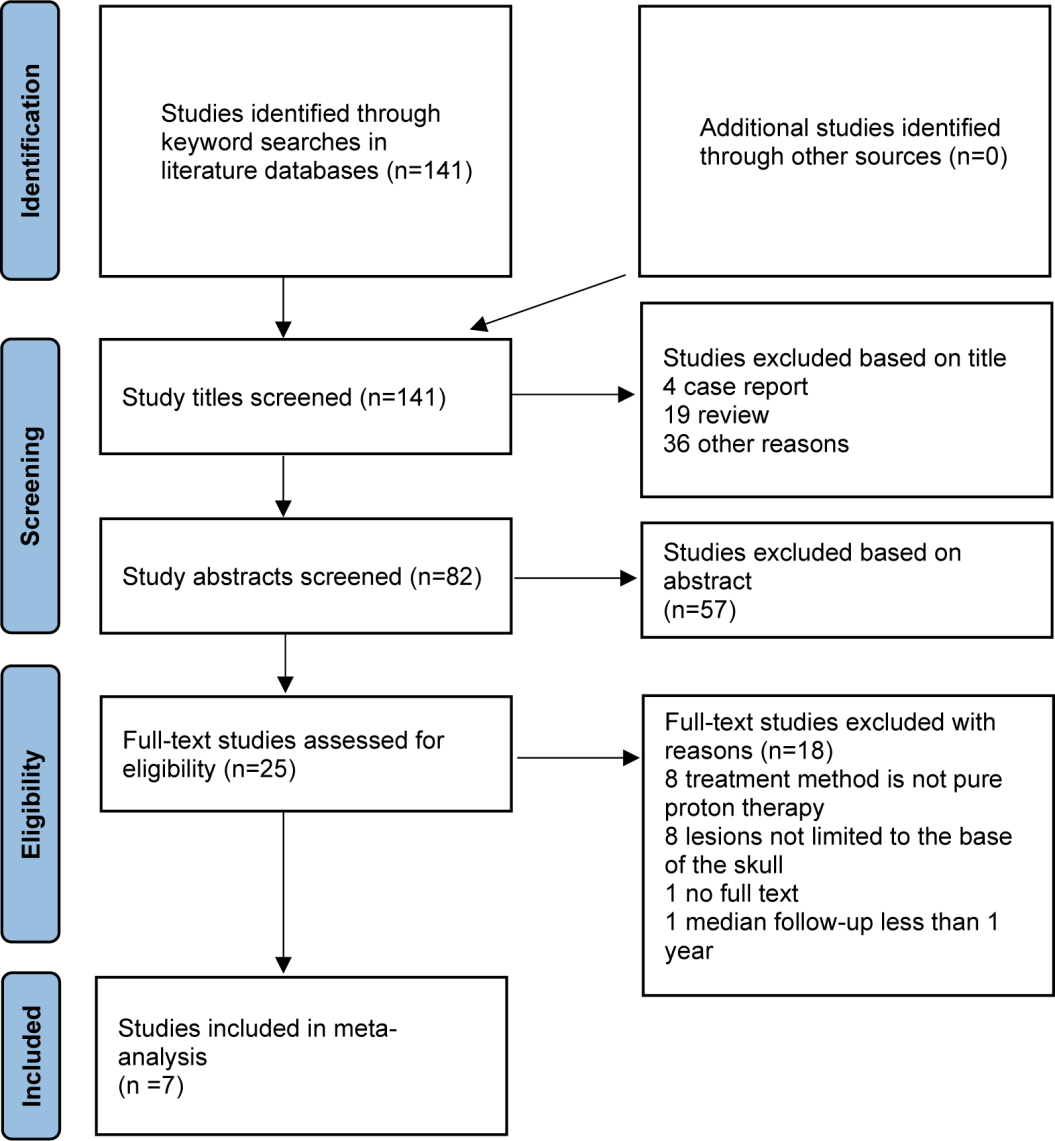
PRISMA flowchart of the literature screening process.

A total of 141 candidate articles were identified from systematic searches of the literature databases, and no additional studies were identified from other sources (**Figure 1**). After screening by title, 4 case reports and 19 reviews were excluded, and 36 studies were inconsistent with our research content. Furthermore, by reading the abstract, 57 articles were filtered. There were then 25 articles remaining for full-text analysis, of which 8 studies were excluded because the treatment methods were mixed with combined proton–photon therapy, 8 studies were excluded because the tumor location was not limited to the skull base, 1 article was excluded because the full text could not be found, and 1 article was excluded because it failed to show enough survival data records. Finally, only seven articles were included in this meta-analysis: one came from the Radiological Research Center of Russia (23), two studies originated from different institutes in Japan (24, 25), two studies were conducted by the Paul Scherrer Institute in Switzerland (26, 27), and two studies were finished by different cancer centers in the USA (28, 29). Of note, one study was specific to chordoma (25), and one study reported only survival outcomes (27).

### Demographics

This study analyzed the clinical data of 478 patients from six centers around the world. Two of the studies did not list the proportions of chordoma and chondrosarcoma separately. In the remaining five studies, there were 211 patients with chordoma and 92 patients with chondrosarcoma. Overall, the median age and follow-up time of the cohort ranged from 38 to 52 years and 21 to 61.7 months, respectively. The proportion of male and female patients in this analysis was about 47% and 53%. The characteristics of the included cohort are summarized in **Table 1**.

**Table 1:** Study characteristics and cohort demographics of all included studies.

Study	Outcomes		Study period	Location	Design	Cohort						
	Survival	Toxicity				Size (*n*)	Chordoma (*n*)	Chondrosarcoma (*n*)	Median age (years)	Female (*n*)	Male (*n*)	Median follow-up (months)
Fuji, H. (2011)	Survival	Toxicity	June 2003–November 2008	Japan	ROCS	16	8	8	38 (9–78)	6	10	42 (9–80)
Deraniyagala, R.L. (2014)	Survival	Toxicity	2007–2011	USA	ROCS	33	NR	NR	NR	7	26	21 (3–58)
Grosshans, D.R. (2014)	Survival	Toxicity	June 2010–August 2011	USA	ROCS	15	10	5	43 (25–69)	10	5	27 (13–42)
Hayashi, Y. (2016)	Survival	Toxicity	February 2006–May 2013	Japan	ROCS	19	19	0	52 (13–76)	11	8	61.7 (31.5–115.4)
Weber, D.C. (2016)	Survival	Toxicity	1998–2012	Switzerland	ROCS	222	151	71	40.8	105	117	50 (4–176)
Hottinger, A.L. (2020)	Survival	NR	2004–2016	Switzerland	ROCS	142	NR	NR	42 (1–79)	66	76	52 (3–152)
Gordon, K. (2021)	Survival	Toxicity	2016–2020	Russia	ROCS	31	23	8	50 (27–71)	20	11	21 (4–52)

### Clinical features

All patients included in the analysis were histopathologically diagnosed with chordoma or chondrosarcoma by gross total resection (GTR) or subtotal resection (STR) or biopsy, among which the proportion of recurrent disease was 6.7% to 36.8%. One of the studies did not clearly report the extent of surgical resection for each patient (24), the proportions who underwent GTR and STR procedures in the remaining six studies were approximately 13% and 83.6%, respectively, and only about 3.4% of the patients received histological biopsies. The clinical features of all included studies are summarized in **Table 2**.

**Table 2:** Clinical features of all included studies.

Study	Pathology	Surgical resection	Diagnostic resection (%)	Recurrent disease (%)	Median target volume (cc)	Proton radiotherapy regimen		
						Total dose (Gy_RBE_)	Fractions (*n*)	Dose/fraction (Gy_RBE_)
Fuji, H. (2011)	Yes	NR	100%	NR	40 (7–546) (GTV)	63 (50–70)	NR	1.8
Deraniyagala, R.L. (2014)	Yes	GTR 27%; STR 67%; Biopsy only 6%	100%	9%	NR	74 (70–79)	NR	1.8–2.0
Grosshans, D.R. (2014)	Yes	GTR 20%; STR 80%	100%	6.70%	15–26.2	69.8 (68–70) chordoma; 68.4 (66–70) chondrosarcoma	NR	1.8–2.0
Hayashi, Y. (2016)	Yes	GTR 42.1%; STR 47.3%; Biopsy only 10.6%	100%	36.80%	NR	9 cases: 77.44; 10 cases: 78.4	77.44/64 bid; 78.4/56 bid	1.2–1.4
Weber, D.C. (2016)	Yes	GTR 3.2%; STR 96.8%	100%	23%	35.7 ± 29.1 (GTV)	72.5 ± 2.2	NR	1.8–2.0
Hottinger, A.L. (2020)	Yes	GTR 13%; STR 83%; Biopsy4%	100%	24%	26.3 (0.0–130.4) (GTV)	74.0 (72.6–80.0)	NR	1.8–2.5
Gordon, K. (2021)	Yes	GTR 32.2%; STR 48.4%	80.6%	12.90%	25.6 (4.2–115.6) (GTV)	70 (60–74)	NR	2

### Proton radiotherapy

For PT, the median target volume ranges from 15 to 40 cc, and the median total dose for proton radiotherapy regimen varies from 63 to 78.4 Gy_RBE_, most of which with a single fraction dose of 1.8–2.0 Gy_RBE_ (**Table 2**). For one of the studies conducted in Japan, Hayashi et al. (25) evaluated the hyperfractionated high-dose proton beam therapy for patients with clival chordomas; the prescribed dose for the first 9 consecutive patients was 77.44 Gy_RBE_ in 64 fractions, and the latter 10 patients were treated with 78.4 Gy_RBE_ in 56 fractions. The dose per fraction was set at 1.2 Gy_RBE_ and 1.4 Gy_RBE,_ respectively. Survival analysis showed that the 5-year LC, cause-specific, and OS rates of the latter 10 cases were higher than all 19 cases, indicating that a hyperfractionated high-dose scheme after maximum surgical resection seems to be efficient for patients with clival chordomas. For another study conducted in Paul Scherrer Institute, Switzerland, Hottinger et al. (27) utilized a total dose of 72.6–80.0 Gy at 1.8–2.5 Gy per fraction.

### Local control

**Figure 2:**
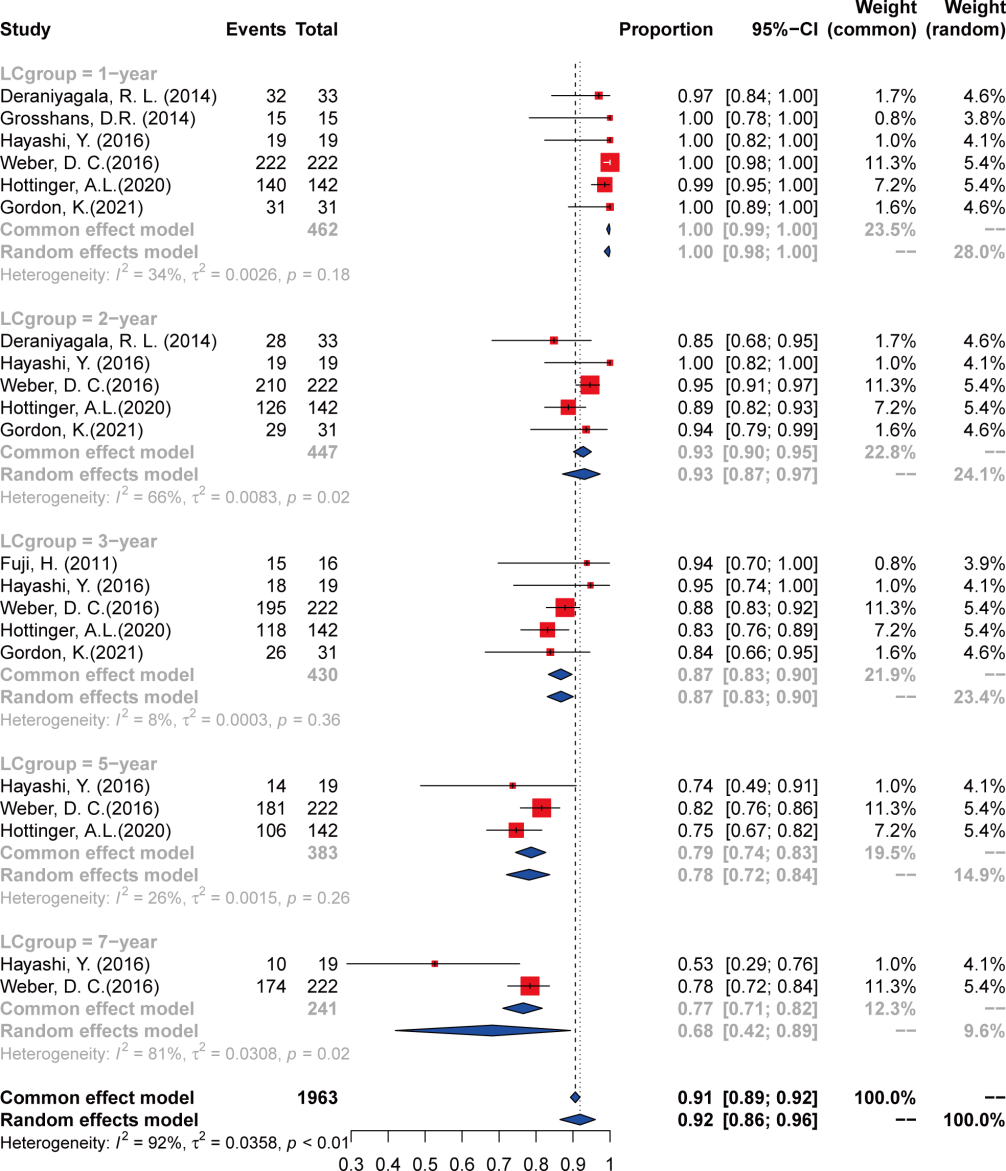
The local control of chordoma and chondrosarcoma treated with PT.

Data were extracted from seven included studies for LC analysis (**Figure 2** and **Table 3**). For the 1-year LC, six studies (23, 25–29) were analyzed, and the incidence was 100% (95% CI 98–100%, *I* ^2^ = 34%). Five studies (23–28) were included in the analysis of the 2-year and 3-year LC rates, with an incidence of 93% (95% CI 89%–97%, *I* ^2^ = 56%) and 87% (95% CI 84%–91%, *I* ^2^ = 0%), respectively. The incidence of 5-year LC was 78% (95% CI 72%–84%, *I* ^2^ = 24%), calculated from three articles (25–27). As to the 7-year LC rate, only two studies (25, 26) were analyzed, and the incidence was 68% (95% CI 43%–93%, *I* ^2^ = NA).

**Table 3:** Local control and overall survival incidence and error estimates as indicated by the contemporary literature, with certainty assessed for each outcome per the GRADE criteria.

All studies (*n* = 478 patients)	Local Control						Overall Survival					
	Number at risk (*n*)	Incidence (%)	95% CI	*I*² (%)	Studies (*n*)	Certainty	Number at risk (*n*)	Incidence (%)	95% CI	*I*² (%)	Studies (*n*)	Certainty
1 year	459	100	98–100	34	6	Moderate	114	100	98–100	0	5	Moderate
2 years	412	93	89–97	56	5	Moderate	96	99	94–100	64	4	Low
3 years	372	87	84–91	0	5	Moderate	55	89	70–100	83	3	Very low
5 years	301	78	72–84	24	3	Very low	326	85	82–89	0	3	Very low
7 years	184	68	43–93	NA	2	Very low	184	68	43–93	NA	2	Very low

### Overall survival

**Figure 3:**
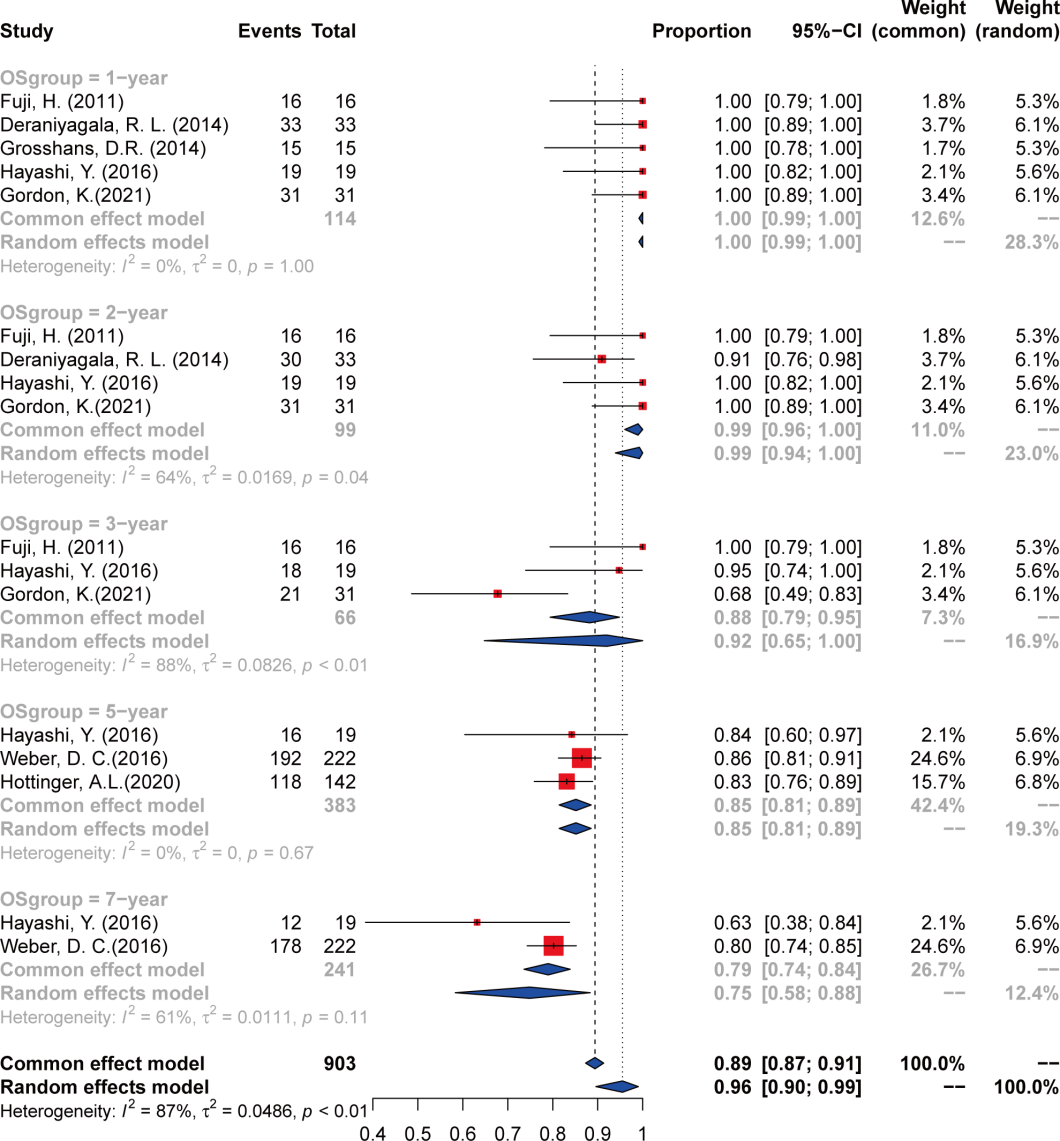
The overall survival of chordoma and chondrosarcoma treated with PT.

The outcomes of OS were calculated from a total of seven studies, including 1-, 2-, 3-, 5-, and 7-year incidence and 95% CI (**Figure 3** and **Table 3**). The 1-year OS was 100% (95% CI 98%–100%, *I* ^2^ = 0%) calculated from five articles (23–25, 28, 29). For the 2-year OS, four studies were analyzed and the incidence was 99% (95% CI 94%–100%, *I* ^2^ = 64%). Analysis of 3- and 5-year OS included three studies (23–27), and the incidence was 89% (95% CI 70%–100%, *I* ^2^ = 83%) and 85% (95% CI 82%–89%, *I* ^2^ = 0%), respectively. As to the 7-year OS, only two studies (25, 26) were analyzed, and the incidence was 68% (95% CI 43%–93%, *I* ^2^ = NA).

### Toxicity

For the seven studies included in the analysis, only one study did not report toxicity (27), and none of the remaining studies reported early grade 3 or higher toxicity. As to the late grade 3 or higher toxicity, two studies were involved. Weber et al. (26) showed that high-grade late toxicity was observed in 8.1% of the overall patients, which consisted of grade 3 unilateral optic neuropathy in 5 (25%) patients, grade 3 temporal lobe necrosis and grade 3 cerebellum brain parenchyma necrosis in 13 (56%) patients and 1 (8%) patient, respectively, grade 3 unilateral hearing loss in 3 (12%) patients, grade 4 bilateral optic neuropathy in 2 (8%) patients, and 1 (8%) patient with grade 4 spinal cord necrosis. Gordon et al. (23) reported two cases of ≥ grade 3 late toxicity, with one case of grade 3 myelitis (11 months after PT) and one case of grade 5 brainstem injury. According to the included studies, reported early or late toxicities of less than grade 3 included otitis media, temporal lobe necrosis, diplopia, unilateral hearing loss, fatigue, vomiting, headache, mild cognitive and memory impairment, keratitis, and laryngeal mucositis.

### Quality assessment and publication bias

The quality of the included articles was assessed according to the NOS (**Supplementary Table 1**). All of the studies were considered moderate to high quality. GRADE assessment results showed that the certainty of the evidence was very low to moderate (**Supplementary Table 2**), mainly due to the high heterogeneity between studies and wide confidence intervals. For the data used for survival analysis, we firstly verified with several methods, including PRAW, PLN, PLOGIT, PAS, and PFT, to ensure that the data conformed to normal distribution. The funnel plots appeared symmetrical in comparison models except the 2- and 3-year OS (**Supplementary Figure 1**), mainly due to high heterogeneity. Sensitivity analysis was performed on the 2- and 3-year OS analysis, and the included articles were separately excluded for re-analysis. Results showed that when the article by Deraniyagala (2014) was excluded, the *I* ^2^ for the 2-year OS decreased to 0, and when the article of Gordon (2021) was excluded from the analysis, *I*² decreases to 0 for the 3-year OS.

## Discussion

The main treatment of chordoma and chondrosarcoma is surgery, and the extent of surgical resection is often related to survival outcomes. However, GTR is hard to achieve. Thus, compared with surgery alone, residual tumors have been treated with adjuvant local radiation therapy. A retrospective review of 20 patients diagnosed with clival chordoma indicated that adjuvant RT after GTR may play a vital role in the elevated progression-free survival and OS (30). However, there remains heterogeneity in literature on the effects of different radiotherapy modalities adopted in skull base chordoma and chondrosarcoma. A meta-analysis indicated that adjuvant radiotherapy improved the survival of patients undergoing partial resection, and the 5-year OS of patients treated with surgery followed by adjuvant radiotherapy was 90% compared with 70% of those treated by surgery alone, but it failed to show significant statistical benefits in this subgroup (31).

To date, apart from conventional photon radiotherapy (CRT), various radiation techniques have been applied for chordoma and chondrosarcoma, such as stereotactic radiosurgery or hypofractionated stereotactic radiotherapy (SRS/SRT), proton therapy (PT), and other heavy charged particles like carbon ion radiotherapy (CIRT), aiming to improve clinical efficacy. However, a previous systematic review showed no difference in 5-year PFS and OS between the various particle types of radiotherapy (32). Thus, the role of radiation therapy for skull base chordomas should be well established.

SRS/SRT could be performed in skull base chordoma by delivering a relatively high and homogenous dose to target volume. An international multi-institutional study analyzed 93 patients who underwent single-session SRS for intracranial chordoma. The mean margin and maximum doses utilized were 17 Gy and 34.2 Gy, respectively. The 5- and 10-year OS rates could reach 83% and 70%, respectively (33). Other articles reported that a better LC can be reached by a higher prescription dose of at least more than 20 Gy (34). However, the adoption of SRS/SRT is usually limited by tumor volume, which made it more appropriate for a small tumor of less than 10 cc to remain after surgery to avoid severe toxicities.

Carbon ion radiotherapy (CIRT) is an emerging radiation modality used in skull base tumor. Lu et al. conducted a meta-analysis of nine studies including 632 patients with skull base chordoma or chondrosarcoma. This research showed favorable results concerning survival outcomes. For chordoma-only studies, the LC incidence at 1, 5, and 10 years was 99%, 80%, and 56%, respectively. As for the 1-, 5-, and 10-year OS probability, the estimated results were 100%, 94%, and 78%, respectively (35). The existing articles showed a low incidence of severe early and late toxicity (grade ≥ 3), ranging from 0 to 4%. However, this new treatment pattern can only be accessible at a limited number of centers around the world.

PT is increasingly becoming the preferred treatment modality because of its unique ballistic characteristics of high-energy particles that allow dose escalation to the tumor and delivery of substantially lower doses to critical structures compared to other radiation modalities (36). However, most systematic reviews on this respect usually included publications that incorporated patients receiving combined beam planning like proton–photon- based radiotherapy. Since the innate dose distribution characteristics were different between photons and protons, combined radiation mode may cause suboptimal dose sparing to the adjacent tissues, which would impair the intended biological effects of the dose-escalated tumor region (37, 38). At the same time, combined mode led to unfavorable protection of the organ at risk, consequently indicating that clinically relevant acute or late toxicities might increase (39). Hence, a systematic review focusing on PT not combined with other radiation modalities can represent one of just a few methods to evaluate the true clinical efficacy of PT on skull base chordoma and chondrosarcoma.

A total of seven retrospective observational cohort studies met the inclusion and exclusion criteria for this analysis and our outcomes were able to load unique metadata to indicate actual clinical efficacy of delivering PT. Herein, we present our results of a systematic review of seven articles concerning PT in a total of 478 patients. Through metadata extraction and analysis, we achieved 100% 1-year LC and OS. The 2- and 3-year LC was 93% and 87%, and 2- and 3-year OS was 99% and 89%, respectively. The symmetry of the funnel plots for 2- and 3-year OS was poor, and sensitivity analysis results indicated that the heterogeneity stemmed from the publications by Deraniyagala (2014) and Gordon et al. (2021). It was believed that the possible reasons were the small number of enrolled patients, and the relatively lower proportion of patients undergoing GTR surgery compared with other involved literatures. With respect to long-term clinical analysis, LC and OS over 5 years were the major concern. Three included articles reported 5-year LC and OS (25–27), and we determined the corresponding incidence to be 78% and 89%, respectively. Only two articles documented the 7-year OS rate (25, 26), and the calculated incidence was 68%. Because of the limited number of involved studies, further analysis between the two pathologic subgroups was not conducted. However, as a whole, our study was consistent with different institutional findings concerning PT, with 5-year OS rates ranging from 62% to 88% for chordoma and 91% to 100% for chondrosarcoma (28, 40–43). A recent meta-analysis revealed that PT was more effective following surgery for chordoma than CRT and confirmed the benefit of PT in chordoma, which showed an OS advantage for protons over conventional radiotherapy at 3 (89% *vs*. 70%), 5 (78% *vs*. 46%), and 10 years (60% *vs*. 21%) (44). A retrospective study of the NCDB also demonstrated improved 5-year OS to be associated with the PT for definitive radiotherapy (45). For long-term survival, typically after 5 years, PT seems to be more beneficial than other radiation management.

Though dose escalation for skull base chordoma and chondrosarcoma shows superiority in survival aspects, it may also be latently treacherous on some critical adjacent structures, specifically the anterior optic pathway. Visual toxicity is an irreversible severe late complication induced by a higher maximum dose (Dmax), among which vision loss is the most detrimental outcome (46). Alexandra et al. (47) analyzed 148 patients and 283 individual eyes with functional vision at baseline receiving a minimum of 30 Gy_RBE_ to 0.1 cm^3^ of the anterior optic pathway. The reported median time to vison loss was 15.2 months following high-dose proton-based radiotherapy. The 5-year incidence of functional blindness was 2.1%. It further demonstrated the acceptable long-term complication of vision loss with an incidence rate of 3.6% over 60 Gy_RBE_ and no blindness occurred under 60 Gy_RBE_. As to our systematic review, Weber et al. (26) showed that high-grade late toxicity was observed in 8.1% of the overall patients, with grade 3 unilateral optic neuropathy occurring in 2.3% of the patients. No late grade 3 or higher toxicities were found in most of the other involved studies. Feuvret et al. (39) compared treatment planning between combined photon–proton planning and proton planning for skull base tumors, so as to assess the potential limitations of combined planning for these tumors. In this research, mean doses delivered to the GTVs and CTVs were not significantly different between the two groups. However, the dose inhomogeneity was drastically increased with the combined beam group. In the proton-only planning, it could significantly reduce the tumor dose inhomogeneity and the delivered sharp kinetic energy to adjacent normal tissues. This may give us a hint that proton-only modality is more suitable for children or patients with longer life expectations, which might achieve better protection against radiation-induced functional impairment and improve quality of life.

Our systematic review also has some limitations that need to be addressed in future studies. In the first place, since chordoma is a slowly progressive bone tumor with a relatively long natural history, the involved seven studies did not have follow-up statistics for more than 7 years, which prompted us to figure out the long-term oncologic outcomes such as recurrence pattern and radiation-induced secondary malignancies. Second, greater cohort studies or prospective studies were encouraged, which would provide us with a more reliable and comprehensive understanding of the management of PT. Last but not least, neurocognitive evaluation and other neuro-oncological relative outcomes, such as quality of life, should be included, since such information is critical to distinguish PT from other radiation modalities so that we could make the best individual recommendations to patients.

## Conclusions

Our analyses have shown the satisfactory survival outcomes and acceptable toxicity of PT. PT might be a promising option to treat chordomas and chondrosarcomas especially for the skull base location. However, currently, a major concern in the use of PT is the limited literature, most of which are retrospective observational cohort studies. With this concern, multicenter randomized controlled studies and prospective clinical trials should be conducted in the future to truly validate the existing outcomes to date. Standardized procedures and observational endpoints of PT in the skull base chordomas and chondrosarcomas are encouraged so as to establish a clear guideline to select the optimal method that is most beneficial to patients.

## Data availability statement

The original contributions presented in the study are included in the article/**Supplementary Material**. Further inquiries can be directed to the corresponding author.

## Author contributions

Conception and design: XQ and MN. Search and collection of data: MN and LC. Data analysis and interpretation: MN and JZ. Manuscript writing: MN and LC. MN and LC contributed equally to this article. All authors contributed to the article and approved the submitted version.

## Conflict of interest

The authors declare that the research was conducted in the absence of any commercial or financial relationships that could be construed as a potential conflict of interest.

## Publisher’s note

All claims expressed in this article are solely those of the authors and do not necessarily represent those of their affiliated organizations, or those of the publisher, the editors and the reviewers. Any product that may be evaluated in this article, or claim that may be made by its manufacturer, is not guaranteed or endorsed by the publisher.

## Supplementary material

Supplementary Figure 1 The funnel plot of 2- and 3-year overall survival.

## Abbreviations

PT, proton therapy; GTR, gross total resection; STR, subtotal resection; LC, local control; OS, overall survival; NOS, Newcastle–Ottawa Scale; GRADE, Grading of Recommendations, Assessment, Development and Evaluations; CRT, conventional photon radiotherapy; SRS/SRT, stereotactic radiosurgery/stereotactic radiotherapy; CIRT, carbon ion radiotherapy
