# Supplementary figures and images for: Pure proton therapy for skull base chordomas and chondrosarcomas: A systematic review of clinical experience
Source: Front Oncol. 2022 Nov 25;12:1016857. doi: 10.3389/fonc.2022.1016857 (PMC9732011; doi:10.3389/fonc.2022.1016857)

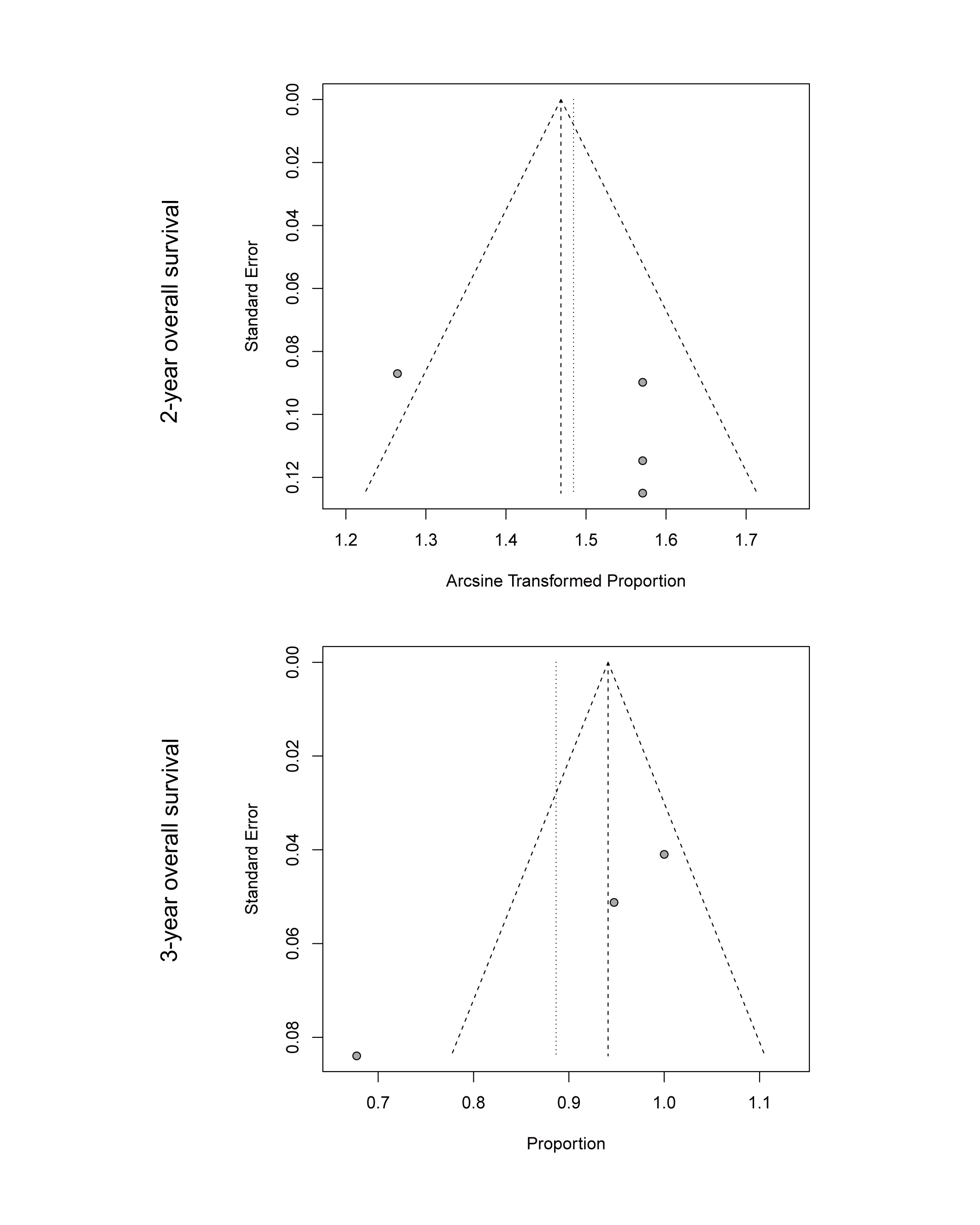

Supplement: Supplementary Figure 1 — The funnel plot of 2- and 3-year overall survival. [file Image_1.tif]
