# Supplementary material for: Pure proton therapy for skull base chordomas and chondrosarcomas: A systematic review of clinical experience
Source: Front Oncol. 2022 Nov 25;12:1016857. doi: 10.3389/fonc.2022.1016857 (PMC9732011; doi:10.3389/fonc.2022.1016857)
Supplement: Supplementary file 2 [file Table_1.docx]

**Supplementary Table 1.** Quality of included studies assessed by NOS criteria.

| **Study** | **Criterion** | | | | | **Total** | **Overall Quality** |
| --- | --- | --- | --- | --- | --- | --- | --- |
|  | **1** | **2** | **3** | **4** | **5** |  |  |
| Fuji, H. (2011) | 1 | 0 | 1 | 1 | 1 | 4 | Moderate |
| Deraniyagala, R. L. (2014) | 0 | 1 | 1 | 0 | 1 | 3 | Moderate |
| Grosshans, D.R. (2014) | 1 | 1 | 1 | 1 | 1 | 5 | High |
| Hayashi, Y. (2016) | 1 | 1 | 1 | 0 | 1 | 4 | Moderate |
| Weber, D. C. (2016) | 1 | 1 | 1 | 1 | 1 | 5 | High |
| Hottinger, A.L. (2020) | 1 | 1 | 1 | 0 | 1 | 4 | Moderate |
| Gordon, K. (2021) | 1 | 1 | 1 | 1 | 1 | 5 | High |

*chordoma only. The items are scored in response to each question as either 1 in the positive, and 0 in the negative/unclear, to lead to a maximum score of 5. Overall quality was assessed based on impression of criteria scoring (low, 0-2; moderate, 3-4; high, 5).
