# Supplementary material for: Pure proton therapy for skull base chordomas and chondrosarcomas: A systematic review of clinical experience
Source: Front Oncol. 2022 Nov 25;12:1016857. doi: 10.3389/fonc.2022.1016857 (PMC9732011; doi:10.3389/fonc.2022.1016857)
Supplement: Supplementary file 3 [file Table_2.docx]

**Supplementary Table 2.** GRADE assessment for reported outcomes. GRADE, Grading of Recommendations, Assessment, Development and Evaluations; NA, not applicable.

| **Outcome** | **Incidence** | **I^2^** | **No. of studies pooled** | **Certainty assessment** | | | | | |  | **Certainty** |
| --- | --- | --- | --- | --- | --- | --- | --- | --- | --- | --- | --- |
|  |  |  |  | **Type of Evidence** | **Quality** | **Consistency** | **Directness** | **Effect size** | **Overall Quality** | |  |
| **Local control** |  |  |  |  |  |  |  |  |  | |  |
| **All studies** |  |  |  |  |  |  |  |  |  | |  |
| 1-year | 100% | 34% | 6 | +2 | -1 | +1 | -1 | +2 | +3 | | Moderate |
| 2-year | 93% | 56% | 5 | +2 | -1 | +1 | -1 | +2 | +3 | | Moderate |
| 3-year | 87% | 0% | 5 | +2 | -1 | +1 | -1 | +2 | +3 | | Moderate |
| 5-year | 78% | 24% | 3 | +2 | -2 | 0 | -1 | +1 | 0 | | Very Low |
| 10-year | 68% | NA | 2 | +2 | -2 | -1 | -1 | 0 | -2 | | Very Low |
| **Overall survival** |  |  |  |  |  |  |  |  |  | |  |
| **All studies** |  |  |  |  |  |  |  |  |  | |  |
| 1-year | 100% | 0% | 5 | +2 | -1 | +1 | -1 | +2 | +3 | | Moderate |
| 2-year | 99% | 64% | 4 | +2 | -1 | 0 | -1 | +2 | +2 | | Low |
| 3-year | 89% | 83% | 3 | +2 | -2 | 0 | -1 | +2 | +1 | | Very Low |
| 5-year | 85% | 0% | 3 | +2 | -2 | 0 | -1 | +2 | +1 | | Very Low |
| 10-year | 68% | NA | 2 | +2 | -2 | -1 | -1 | 0 | -2 | | Very Low |

The overall quality score is determined based on the sum of the included domains. Type of evidence is based on design of the included studies (range, +2 to +4). The study quality reflects the blinding and allocation, follow-up and withdrawals, sparsity of data, and methodological concerns (range, -3 to 0). Consistency is graded based on heterogeneity of included population and study end points with respect to one another (range, -1 to +1). Directness is graded based on generalizability of included results (range, -2 to 0). Effect size is graded based on pooled incidence overlaps with first and tenth decile (range, 0 to 2). The overall quality of results for each outcome can be considered high (≥ 4 points), moderate (3 points), low (2 points) or very low (≤ 1 point).
